# Cardiovascular Autonomic Dysfunction in Patients with Morbid Obesity

**DOI:** 10.5935/abc.20150125

**Published:** 2015-12

**Authors:** Maurício de Sant Anna Junior, João Regis Ivar Carneiro, Renata Ferreira Carvalhal, Diego de Faria Magalhães Torres, Gustavo Gavina da Cruz, José Carlos do Vale Quaresma, Jocemir Ronaldo Lugon, Fernando Silva Guimarães

**Affiliations:** 1 Programa de Tratamento Multidisciplinar da Obesidade do Hospital Universitário Clementino Fraga Filho da Universidade Federal do Rio de Janeiro - UFRJ - Rio de Janeiro, RJ - Brazil; 2 Departamento de Fisioterapia do Centro Universitário Anhanguera Niterói - UNIAN, Niterói, RJ - Brazil; 3 Divisão de Nefrologia - Faculdade de Medicina da Universidade Federal Fluminense - UFF, Niterói, RJ - Brazil; 4 Departamento de Fisioterapia da Universidade Federal do Rio de Janeiro - UFRJ, Rio de Janeiro, RJ - Brazil; 5 Programa de pós-graduação em Ciências Médicas, Universidade Federal Fluminense - UFF, Niterói, RJ - Brazil; 6 Instituto Federal de Educação, Ciência e Tecnologia do Rio de Janeiro, Rio de Janeiro, RJ - Brazil; 7 Programa de pós-graduação em Ciências da Reabilitação - Centro Universitário Augusto Motta, Rio de Janeiro, RJ - Brazil

**Keywords:** Obesity, Morbid, Cardiovascular Diseases, Risk Factors, Pulmonary Heart Disease / complications, Heart Rate

## Abstract

**Background:**

Morbid obesity is directly related to deterioration in cardiorespiratory capacity, including changes in cardiovascular autonomic modulation.

**Objective:**

This study aimed to assess the cardiovascular autonomic function in morbidly obese individuals.

**Methods:**

Cross-sectional study, including two groups of participants: Group I, composed by 50 morbidly obese subjects, and Group II, composed by 30 nonobese subjects. The autonomic function was assessed by heart rate variability in the time domain (standard deviation of all normal RR intervals [SDNN]; standard deviation of the normal R-R intervals [SDNN]; square root of the mean squared differences of successive R-R intervals [RMSSD]; and the percentage of interval differences of successive R-R intervals greater than 50 milliseconds [pNN50] than the adjacent interval), and in the frequency domain (high frequency [HF]; low frequency [LF]: integration of power spectral density function in high frequency and low frequency ranges respectively). Between-group comparisons were performed by the Student’s t-test, with a level of significance of 5%.

**Results:**

Obese subjects had lower values of SDNN (40.0 ± 18.0 ms vs. 70.0 ± 27.8 ms; p = 0.0004), RMSSD (23.7 ± 13.0 ms vs. 40.3 ± 22.4 ms; p = 0.0030), pNN50 (14.8 ± 10.4 % vs. 25.9 ± 7.2%; p = 0.0061) and HF (30.0 ± 17.5 Hz vs. 51.7 ± 25.5 Hz; p = 0.0023) than controls. Mean LF/HF ratio was higher in Group I (5.0 ± 2.8 vs. 1.0 ± 0.9; p = 0.0189), indicating changes in the sympathovagal balance. No statistical difference in LF was observed between Group I and Group II (50.1 ± 30.2 Hz vs. 40.9 ± 23.9 Hz; p = 0.9013).

**Conclusion:**

morbidly obese individuals have increased sympathetic activity and reduced parasympathetic activity, featuring cardiovascular autonomic dysfunction.

## Introduction

The prevalence of obesity, which is considered an alarming public health problem in the world, has increased dramatically in recent years and become an epidemic^1,2^, including in Brazil^3^. Obesity has a multifactorial etiology that encompasses nutritional, genetic, psychic, socioeconomic factors and sedentary lifestyle^1-3^. Excess body weight is associated with cardiovascular, cerebrovascular, respiratory, metabolic and oncologic diseases^4-7^.

Obesity may be classified using the Body Mass Index (BMI); a BMI varying from 30 kg/m^2^to 34.9 kg/m^2^ is classified as class I obesity, 35 kg/m^2^to 39.9 kg/m^2^ as class II obesity, and a BMI ≥ 40 kg/m^2^as class III obesity, also known as morbid obesity^1,4^. Some authors suggest the inclusion of further categories, a BMI ranging from 30 kg/m^2^to 34.9 kg/m^2^ for super-obese, BMI ≥ 60 kg/m^2^ for super-super obese^8^.

Morbid obesity is directly associated with deterioration of cardiorespiratory capacity, leading to reduction of pulmonary capacity and functional residual capacity^9,10^, hypoventilation syndrome^11,12^, obstructive sleep apnea^13^, increased respiratory muscle strength^14^, and changes in the autonomic function^15,16^.

Assessment of heart rate variability (HRV) quantifies the oscillations in the interval between consecutive heartbeats (R-R intervals), and oscillations between consecutive instantaneous heart rates. HRV may be evaluated either in short or long periods, and its main advantage is the selectivity and non-invasiveness in assessing the cardiovascular autonomic function^17,18^.

Changes in the autonomic modulation, particularly the reduction of HRV, are risk factors for sudden death in conditions like post-acute myocardial infarction and heart failure^19,20^. Changes in HRV responses are a valuable, early indicator of impairment of cardiovascular health.

The hypothesis of this study was that the cardiovascular autonomic function is affected by obesity and becomes an additional cardiovascular risk in this population^21-23^. The aim of this study was to assess the cardiovascular autonomic function in morbidly obese individuals.

## Methods

This was a cross-sectional study on 80 subjects aged from 20 to 60 years recruited in the Bariatric Surgery Program of the Clementino Fraga Filho University Hospital, Federal University of Rio de Janeiro (PROCIBA - HUCFF / UFRJ). Subjects were divided into two groups, Group I, composed of 50 morbidly obese individuals, and Group II, composed of 30 nonobese individuals, matched for age and height. All participants signed an informed consent document, according to the Brazilian National Council for Health (resolution number 466/12). The study was approved by the institutional research ethics committee (Comitê de Ética em Pesquisa do HUCFF-UFRJ, number 077/09).

The following exclusion criteria were adopted: hemodynamic instability at evaluation, heart failure (identified by the two-dimensional transthoracic echocardiography), obstructive pulmonary disease (forced expiratory volume in the first second [FEV1]/forced vital capacity [FVC] < 70% and FEV1 < 70% of predicted), smoking, history of sleep apnea and/or diurnal hypersomnolence, measured by the Epworth scale^24^. Anthropometric assessment was performed by measures of body weight (using an InBody 230, Biospace, Seoul, Korea), height, BMI, and waist-to-hip ratio (WHR)^25^.

### Forced Spirometry

Spirometry was performed according to the American Thoracic Society^26^ and the Brazilian Society of Pneumology^27^guidelines, using a computerized spirometer and its components, including a Lilly-type pneumotachograph (Erich Jaeger, Hoechberg, Germany), volume and flow transducers (Sensym SLP004D, Honeywell Sensing and Control, Golden Valley, MN, USA), following the manufacturers’ protocol. Predicted values were calculated using the equations proposed by Pereira et al^28^.

### Assessment of static respiratory pressures

Assessment of respiratory muscle strength was conducted by measurements of maximal inspiratory and expiratory pressures (IP_max_ and EP_max_ respectively), according to the methods described by Black & Hyatt^29^. An aneroid mannometer/vacuometer (M120 - Comercial Médica - São Paulo - Brazil) and a mouthpiece containing a 2 mm-hole aiming to dissipate pressures generated by facial and oropharyngeal muscles were used. Three measures were obtained from each participant, with a 2-min interval between them, and the best measures obtained in both groups were considered for analysis. Predicted values were those referred by the Brazilian Society of Pneumology and Tuberculosis Pulmonary Function Test Guidelines^27^.

### Heart rate variability

The cardiovascular autonomic function was assessed by analysis of HRV in the time domain and frequency domain. All subjects were instructed to abstain from coffee, tea, and cola and cocoa beverages for at least two hours prior to the test, and to refrain from physical exercise for twenty-four hours before the test.

Heart rate was recorded under resting condition in sitting position between 8 and 10 o’clock in the morning to avoid influences of the circadian rhythm on heart rate and HRV. A heart rate monitor (S810 - Polar^®^ - Kempele - Finland) was used over a 15-minute period and the beat-to-beat heart rate was recorded through infrared signals^30^. Subjects were also instructed not to talk or move during the acquisition of signs, which was performed in a quiet, silent, temperature controlled (21ºC - 23ºC) room. HRV analysis was performed using the Kubios HRV software, version 2.0 (Kuopio - Finland). For the spectral analysis of HRV, R-R interval time series were analyzed by fast Fourier transform^31^. The first two minutes of the test were not included in calculation of HRV to avoid signal instability and artifacts.

### Analysis of heart rate variability in the frequency domain

Spectral power was calculated by integrating the function of power spectral density in high frequency range (HF: 0.15 - 0.40 Hz) and low frequency range (LF: 0.04 - 0.15 Hz) into normalized units (un). The spectral components were then expressed as the ratio between high frequency range and low frequency range (HF/LF ratio), which reflects the sympathovagal balance^32^.

### Analysis of heart rate variability in the time domain

Analysis of the HRV in the time domain was determined from the RR intervals, using the mean of 5-minute periods or all the monitoring period. A mean of 100 or more successive R-R intervals was considered, and sudden fluctuations > 25% than preceding interval were excluded to exclude extrasystoles from the analysis. The square root of the mean squared differences of successive R-R intervals (RMSSD), the standard deviation of the normal R-R intervals (SDNN), and the percentage of interval differences of successive R-R intervals greater than 50 milliseconds than the adjacent interval (pNN50) were used for analysis^32^.

### Statistical analysis

Sample size was calculated based on the results of the study by Paschoal et al^6^, with a statistical power of 0.8 and significance level of 0.05. Twenty-eight subjects in each group (control and obese) would be needed. The SigmaStat 3.1 software (Jandel Scientific, San Rafael, CA, USA) was used for data analysis and graphs were produced using the SigmaPlot 9.01 software (Jandel Scientific, San Rafael, CA, USA). Data distribution was evaluated by the Shapiro-Wilk test, and group comparisons were performed by the unpaired Student's t test. A p-value < 0.05 was considered statistically significant.

## Results

Characteristics of anthropometry, diurnal somnolence and heart rate in obese and nonobese groups are depicted in Table 1.

**Table 1:** Characteristic of anthropometry, diurnal somnolence and heart rate in the study group (morbidly obese) and control (nonobese)

**Variables**	**Morbidly obese (n = 50)**	**Nonobese (n = 30)**	**p**
Age (years)	40.0 ± 10.4	37.6 ± 11.5	0.2947
Height (m)	1.64 ± 0.09	1.67 ± 0.09	0.3004
Body weight (kg)	138.8 ± 33.6	65.2 ± 10.3	< 0.0001
BMI (kg/m^2^)	50.7 ± 8.9	23.2 ± 2.2	< 0.0001
WC (cm)	136.3 ± 18.8	80.5 ± 9.9	< 0.0001
HC (cm)	143.4 ± 17.5	97.5 ± 5.9	< 0.0001
WHR	0.95 ± 0.09	0.84 ± 0.08	< 0.0001
HR (bpm)	76 ± 13	71 ± 9	0.3269
Epworth	6.8 ± 3.2	7.0 ± 3.5	0.5059

Values in mean ± standard deviation. BMI: Body mass index; WHR: Waist-hip ratio; WC: Waist circumference; HC: Hip circumference; HR: Heart rate.

In the obese group, 54% (n = 27) of individuals were hypertensive under medical treatment, and 16% (n = 8) had diabetes mellitus. Of the patients using medication, 44% (n = 12) used diuretics, 63% (n = 17) used angiotensin-converting-enzyme inhibitor, 22% (n = 6) used beta-blockers, and 75% (n = 20) metformin. No significant differences were observed in pulmonary function between morbidly obese and obese subjects (Table 2), and no participant was diagnosed with pulmonary disease.

**Table 2:** Spirometric variables and maximal static respiratory pressures in morbidly obese and nonobese subjects

**Variables**	**Obese (n = 5D)**	**Nonobese (n = 3D)**	**p**
FVC (% pred)	78.7 ± 12.3	100.9 ± 10.6	0.4198
FEV1 (% pred)	80.5 ± 10.2	97.4 ± 8.0	0.0978
FEV_1_/FVC (%)	85.4 ± 6.2	85.4 ± 9.3	0.2373
EFP (% pred)	83.4 ± 20.3	86.6 ± 13.3	0.5750
MVV (% pred)	89.2 ± 23.4	89.9 ± 15.6	0.3236
PI_max_ (% pred)	100.2 ± 31.5	121.7 ± 25.5	0.0572
PE_max_ (% pred)	107.8 ± 30.5	102.0 ± 11.3	0.2359

FVC: Forced vital capacity; FEV1: Forced expiratory volume in the first second; EFP: Expiratory flow peak; MVV: Maximal voluntary ventilation; PI_max_: Maximal inspiratory pressure; PE_max_: Maximal expiratory pressure. Values in mean ± standard deviation.

**Figure 1:**
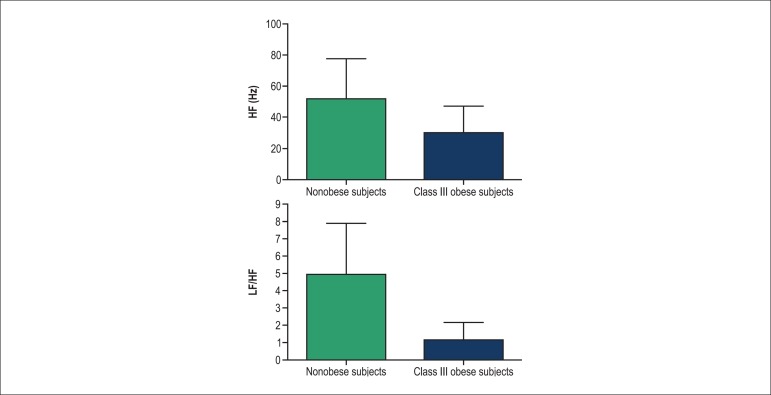
Comparison of heart rate variability in the time domain between morbidly obese and nonobese (control) subjects. HF: High frequency, LF: Low frequency, *p < 0.05.

**Figure 2:**
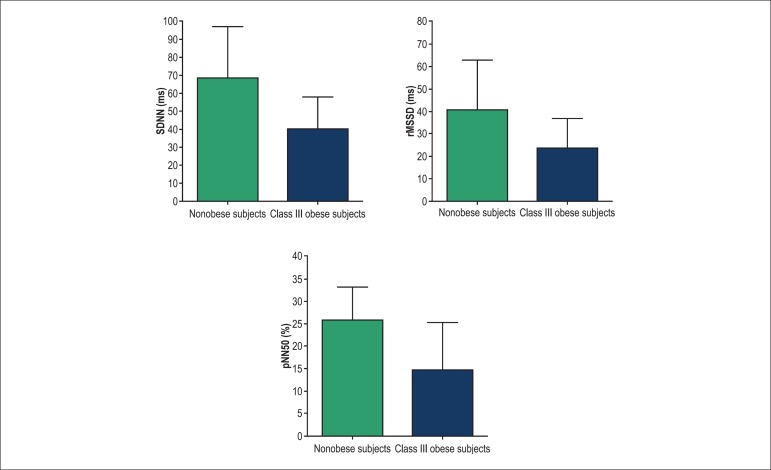
Comparison of heart rate variability in the frequency domain between morbidly obese and nonobese (control) subjects. SDNN: Standard deviation of the normal R-R intervals; RMSDD: Square root of the mean squared differences of successive R-R intervals; pNN50: Percentage of interval differences of successive R-R intervals greater than 50 milliseconds than the adjacent interval; *p < 0.05

**Figure 3:**
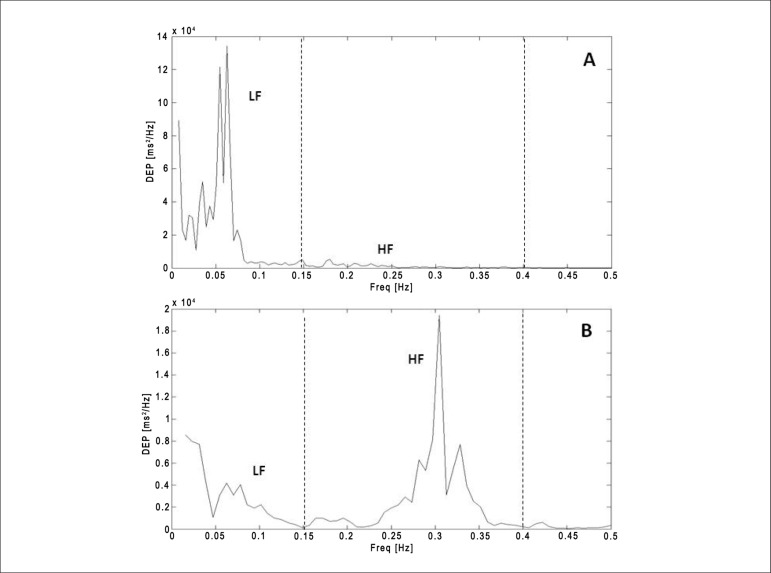
Power spectrum representation of a morbidly obese volunteer (A = 37 years old, 172.6 kg of body weight, 1.78 m of height and body mass index 54.6 kg/m^2^) and a nonobese volunteer (B = 40 years old, 73.4 kg of body weight, 1.73 m of height and body mass index 24.5 kg/m^2^).

Lower values of RMSSD (40.0 ± 18.0 ms *vs.* 70.0 ± 27.8 ms; p = 0.0004), RMSSD (23.7 ± 13.0 ms* vs.* 40.3 ± 22.4 ms; p = 0.0030), pNN5 (14.8 ± 10.4% *vs.* 25.9 ± 7.2%; p = 0.0061) (Figure 1), HF (30.0 ± 17.5 Hz *vs.* 51.7 ± 25.5 Hz; p = 0.0023) and LF/HF ratio (5.0 ± 2.8 *vs.* 1.0 ± 0.9; p = 0.0189) were found in Group I as compared to Group II (Figures 2 and 3). No significant difference was observed in LF values between the groups (50.1 ± 30.2 Hz *vs.* 40.9 ± 23.9 Hz; p = 0.9013).

## Discussion

This study aimed to evaluate the cardiovascular autonomic function in morbidly obese subjects by HRV analysis, and showed an important reduction in the parasympathetic activity in this group of individuals as compared to healthy controls.

Assessment of the HRV in the time domain consists in the acquisition of continuous electrocardiographic recordings during short or long periods to obtain the distribution of intervals between the normal RR intervals. Numerous indexes for HRV measurement have been described in the literature based on statistical, arithmetical and geometrical calculations^7,17,18,32^.

Assessment of HRV in the frequency domain is based on the spectral power analysis, which describes the distribution of density as a function of frequency. This analysis depends on the spectral decomposition of HR into its causing components, which are described in terms of the frequency they affect heart rate. The power spectral density may be calculated by fast Fourier transform algorithms or autoregressive models^17,32^.

Jean-Baptiste Joseph Fourier demonstrated that the signals are generally composed by sinusoidal waves with different widths, phases and frequency response. Also, each periodic signal may be decomposed into its respective waves, hence separating the frequency responses^17,18,32^.

Reduced HRV has been indicated by several researchers as a morbidity and mortality predictor in acute myocardial infarction^33^, heart failure^34^ and pulmonary hypertension^35^. Evidence from the literature indicates that global mortality is 5.3 times higher in individuals with lower HRV (SDNN < 50 ms), quantified by time domain indexes. Additionally, the predictive power of HRV was independent from other factors^36^. In our study, morbidly obese individuals had a low mean SDNN value (40.0 ms). In a cross-sectional study on 25 subjects of both genders, aged 45.1 ± 15.2 years, FVC was different between nonobese individuals (BMI from 20 to 25 kg/m^2^) and those with BMI > 25 kg/m^2^. The authors also found a significantly decrease in the parasympathetic activity, indicated by the domains of HF^16^. These findings are similar to our results supporting an important reduction of HRV in the frequency domain (HF). However, differently from the study by Molfino et al^16^, we did not exclude individuals using cardiovascular drugs, due to elevated BMI of our study group and the need to guarantee their safety.

Several studies are in agreement with our findings^25^. In an investigation on the autonomic cardiovascular function in obesity^21^, obese individuals of both genders, aged 42.7 ± 9.3 years were divided into three groups according to the BMI ranges. The first group was composed by 17 subjects (BMI 27 - 32 kg/m^2^), the second group by 13 subjects (BMI 33 - 40 kg/m^2^), and the third group by 12 subjects (BMI > 40 kg/m^2^). After analysis of HRV in the frequency domain, the authors observed that BMI increased as HF significantly decreased. These findings are also in consonance with our results, although we did not perform the stratification of patients by BMI, since our study groups were composed by morbidly obese and healthy controls only.

Similar findings have been demonstrated by a study conducted by Swiss investigators^15^ evaluating the HRV of normal weight and obese women. Mean age and BMI of the normal weight women were 40.1 ± 2.4 years and 21.5 ± 0.5 kg/m^2^respectively. The obese women were divided into three groups according to their BMI; the first group was composed by women aged 44.4 ± 3.5 and BMI 25 - 30 kg/m^2^, the second group by women aged 42.6 ± 1.9 years and BMI 30 - 40 kg/m^2^, and the third group by women aged 35.2 ± 2.0 years and BMI > 40 kg/m^2^. Higher baseline heart rate and reduced parasympathetic activity (measured in both time and frequency domains) were found in obese women with BMI > 40 kg/m^2^as compared with obese women with lower BMI and nonobese women. These findings are similar to our results, in addition to similarities between the study groups of both studies, including the mean age in the morbidly obese groups (40.0 ± 10.4 *vs.* 37.6 ± 11.5 years). Also, similarly to our study, hypertensive, insulin-resistant obese women were not excluded in the study by Sztajzel J et al^15^. However, morbidly obese subjects in our study had higher BMI (44.2 ± 0.7 kg/m^2^ *vs.* 50.7 ± 8.9 kg/m^2^) and their baseline heart rate was not different as compared to nonobese subjects.

A Polish study^37^ evaluated the cardiac autonomic function by HRV in two groups of patients with acute myocardial infarction with clinical hemodynamic and stability (Killip I-II class, without arrhythmic events and/or ventricular dysfunction). The first group was composed by obese, mean age of 54.06 ± 7.04 years and BMI of 32.0 ± 1.78 kg/m^2^, the second group was composed by nonobese subjects, mean age of 55.26 ± 6.62 years and BMI of 23.63 ± 1.27 kg/m^2^. The time domain indexes of HRV (SDNN, RMSSD and pNN50) were reduced in obese as compared to nonobese subjects. Additionally, analysis of HRV in the frequency domain revealed that LF and LF/HF ratio were elevated, and HF was reduced, with statistical significance. These findings corroborate our results, which indicated reduced parasympathetic activity in both time (SDNN, RMSSD and pNN50) and frequency domains (HF). It is of note that in none of the studies on HRV and morbid obesity here mentioned the pulmonary function was described. In our study, individuals with obstructive changes (FEV1/FVC < 70% and FEV1 < 70% of predicted) were excluded, since airway obstruction is a contributing factor to the increase in sympathetic activity^38-40^.

One of the main limitations of this study is that a polysomnographic study aiming to identify and exclude patients with sleep apnea was not performed. In order to reduce this bias, subjects with diurnal somnolence, assessed by the Epworth questionnaire, were excluded. However, despite this limitation, we believe that the present study makes an important contribution to the literature by adding the reduced HRV to other well-known cardiovascular risk factors associated with obesity^21-23^. Therefore, analysis of cardiac autonomic function by HRV may be a useful tool for cardiovascular risk stratification in morbidly obese individuals. Further studies to investigate the impact of pulmonary function and fat distribution on HRV in morbid obesity should be conducted.

## Conclusion

Morbidly obese individuals have increased sympathetic activity and reduced parasympathetic activity, which features a cardiovascular autonomic dysfunction.
